# Japanese Quince (*Chaenomeles japonica*) as a Potential Source of Phenols: Optimization of the Extraction Parameters and Assessment of Antiradical and Antimicrobial Activities

**DOI:** 10.3390/foods9081132

**Published:** 2020-08-17

**Authors:** Ieva Urbanavičiūtė, Mindaugas Liaudanskas, Česlovas Bobinas, Antanas Šarkinas, Aistė Rezgienė, Pranas Viskelis

**Affiliations:** 1 Biochemistry and Technology Laboratory, Institute of Horticulture, Lithuanian Research Centre for Agriculture and Forestry, Kauno st.30, Babtai, LT-54333 Kaunas distr., Lithuania; ceslovas.bobinas@lammc.lt (Č.B.); biochem@lsdi.lt (P.V.); 2 Laboratory of Pharmaceutical Science, Institute of Pharmaceutical Technologies of the Faculty of Pharmacy of Lithuanian University of Health Sciences, Sukilėlių st.13, LT-50162 Kaunas, Lithuania; mindaugas.liaudanskas@lsmuni.lt; 3 Department of Pharmacognosy, Faculty of Pharmacy, Lithuanian University of Health Sciences, Sukilėlių st.13, LT-50162 Kaunas, Lithuania; 4 Microbiological Research Laboratory, Food Institute of Kaunas University of Technology, Radvilėnų pl. 19, 50292 Kaunas, Lithuania; antanas.sarkinas@ktu.lt (A.Š.); aiste.rezgiene@ktu.lt (A.R.); 5 Department of Food Science and Technology of Kaunas University of Technology, Radvilėnų pl. 19, LT-50254 Kaunas, Lithuania

**Keywords:** *Chaenomeles japonica* fruit, polyphenols, antioxidants, antimicrobial activity

## Abstract

The value of fruits is determined by the quantity and variety of biologically active compounds they contain, and their benefits on human health. This work presents the first study of the biochemical composition and antibacterial activity of the new Japanese quince (JQ) cultivars ‘Darius’, ‘Rondo’, and ‘Rasa’ fruits. The total phenolic content (TPC) was determined using the Folin–Ciocalteu method and each compound was identified by HPLC High Performance Liquid Chromatography. The antimicrobial activity against three Gram-positive and three Gram-negative bacteria, and one yeast strain, was evaluated by the agar well diffusion method using three different concentrations. The free radical scavenging activity was determined using DPPH (2,2-diphenyl-1-picrylhydrazyl) and ABTS (2,2′-azino-bis-3-ethylbenzthiazoline-6-sulphonic acid) methods and ranged from 99.1 to 115.9 μmol_TE_/100 g, and from 372 to 682 μmol_TE_/100 g, respectively. TPC ranged from 3906 to 4550 mg_GAE_/100 g, and five compounds, isoquercitrin, rutin, (+)-catechin, (–)-epicatechin, and chlorogenic acid were identified. All JQ extracts possessed antimicrobial activity against Gram-positive and Gram-negative bacteria, and *Enterococcus faecalis* (ATCC 29212) was the most sensitive strain. These results indicate that JQ fruits are a significant source of bio-compounds, which can enrich the diet with strong antioxidants, and they are very promising as a substitute for chemical preservatives in the food and cosmetic industry.

## 1. Introduction

Japanese quince (*Chaenomeles japonica* (Thunb.) Lindl. ex Spach) is a dwarf shrub that originated in East Asia and was used in Chinese medicine 3000 years ago [1]. Quince of the *Chaenomeles* genus is one of the oldest cultivated plants belonging to the *Rosaceae* family, a subgenus of *Maloideae* [1,2]. Studies of the biological activity of Japanese quince (JQ) fruits have revealed their great potential for human health, including growth promotion of the beneficial intestinal bacteria *Lacticaseibacillus casei* and *Lactiplantibacillus plantarum*, protective effect on the lipid membrane against free radicals, and inhibition of cyclooxygenase involved in the inflammatory reactions [3]. Other researchers have shown that extracts of JQ fruits are promising raw material for cancer treatment and prevention, due to their phenols composition and cytotoxic activity [4,5,6,7].

JQ fruit extracts have strong biological activity due to their particular biochemical composition and content of bio compounds. Due and co-authors established 24 phenolic compounds in five *Chaenomeles* species, their quantity and distribution were different only for chlorogenic acid, catechin, procyanidin B1, epicatechin, and procyanidin B2 [8]. Differences in the antioxidant activity of these five species fruits were observed in the same study [8]. Another study identified eleven phenolic compounds, which were dominated by (−)-epicatechin and procyanidin B2 [3]. Besides that, JQ fruits and their juice have a high amount of ascorbic acid (the main biologically active form of vitamin C), which acts as a biological antioxidant and can contribute to chronic disease prevention [1,9,10]. In addition, a number of dietary fibers and pectin were reported [11,12], which are beneficial in bodyweight control, and could prevent the progression of type 2 diabetes and heart diseases [13].

Phenolic compounds are a large and diverse group of molecules, in which the structural characteristics determine their biological activity. The antioxidant activity of phenols depends on the hydroxyl group number, and their configuration in B-ring [14,15]. Structural differences between phenols cause distinct mechanisms of actions against microorganisms, and consequently, their effectiveness [16].

Numerous studies have shown that the phenolic compounds are promising biologically active compounds that may act as a new type of antimicrobial agent [17,18,19]. Kikowska and co-authors demonstrated the antibacterial activity of JQ leaf and fruit extracts against four bacteria strains *Staphylococcus aureus* (ATCC 25923), *Escherichia coli* (ATCC 25922), *Pseudomonas aeruginosa* (ATCC 27853), and one yeast strain *Candida albicans* (ATCC 10231) [20]. The antibacterial activity of other species such as *Chaenomeles speciosa* essential oil against 10 microorganisms has been studied [21]. However, a limited number of studies have reported the antibacterial activity of *Chaenomeles japonica* species fruits.

The extraction efficiency of phenols depends on many conditions, including the solvent system, extraction time, temperature, ultrasound power, etc. [22,23,24]. Response surface methodology (RSM) is a convenient tool to estimate several variables and their interaction influence on total phenolic content (TPC), and optimize the extraction conditions [25,26].

Currently, the cultivation of JQ is gaining popularity in northern European countries, especially in the Baltic Sea area [27]. JQ is very diverse in plant and fruit characteristics, and its propagation by the seeds can cause morphological and biochemical heterogeneity. Breeding new cultivars change the genetic context and leads to morphological, physiological, and metabolic variations [28]. Within the project “Japanese Quince—A new European fruit crop for the production of juice, flavor, and fiber” from 1998–2001, the thornless cultivars named ‘Darius’, ‘Rondo’, and ‘Rasa’ were released. The differences of the bio-compounds composition in leaves and seed oils of these cultivars were studied [29,30,31]. Nevertheless, the biochemical composition and biological activity of their fruits have not yet been investigated.

This study aimed to optimize the phenols extraction conditions, determine the biochemical composition, antiradical, and antibacterial activity of Japanese quince cultivars ‘Darius’, ‘Rondo’, and ‘Rasa’, cultivated in Lithuania.

## 2. Materials and Methods

### 2.1. Plant Material

Fresh Japanese quince fruits (cvs. Darius, Rondo, and Rasa) were obtained from the garden of the Institute of Horticulture, Lithuanian Research Center for Agriculture and Forestry, Babtai (55°60′ N, 23°48′ E) Lithuania in 2018. The fruits were cut into slices, and lyophilized with a ZIRBUS sublimator 3 × 4 × 5/20 (ZIRBUS technology, Bad Grund, Germany) at the pressure of 0.01 mbar (condenser temperature, −85 °C). The slices were ground to powder by using a knife mill GRINDOMIX GM 200 (Retsh, Haan, Germany).

### 2.2. Maceration Extraction Method

First, 0.5 g of the powdered sample with 10 mL solvent in different concentrations (ratio 1:20, *w*/*v*) were mixed and left in the dark for 24 h at room temperature 22 °C. Then, the mixtures were centrifuged and filtered through a Whatman filter paper. Three different solvents (ethanol, methanol, and acetone) and three concentrations of each solvent (100%, 70%, and 50%) were used for the maceration extraction.

### 2.3. Ultrasound Extraction Method and Experimental Design

First, 0.5 g of the powdered sample was mixed with 10 mL 50% ethanol. The ultrasound extraction (UE) of phenolic compounds carried out using a Sonorex Digital 10 P ultrasonic bath (Bandelin Electronic GmbH & Co. KG, Berlin, Germany). Response surface methodology (RSM) was used to examine the influence of UE processing variables on phenols extraction. The impact of three factors (ultrasound power, extraction time, and temperature) on the response (TPC) was modeled according to a central composite design. Ultrasonic power ranged from 48 to 480 W and chosen according to the limitations of the ultrasonic device. The selected extraction temperature did not exceed 60 °C to avoid the degradation of compounds. The experimental design of the three-level-three-factor was composed; consisting of twenty experimental runs including six replicates at the center point. Design-Expert 7 (Stat-Ease Inc., Minneapolis, MN, USA) software was used for statistical analysis of the obtained data. The experimental results fit a first-order polynomial model to obtain the regression coefficients by Equation (1):

Y = β_0_ + β_1_*X*_1_ + β_2_*X*_2_ − β_3_*X*_3_, (1)

where Y is the predicted response (TPC), *X*_1_, *X*_2_, and *X*_3_ meet the variables namely ultrasonic power, extraction time, and temperature, respectively. The β_0_, β_1_, β_2_, and β_3_ values represent their corresponding regression coefficients.

Design-Expert 7 software was used to draw up 3D response surface plots. To estimate the statistical significance of the proposed model, Fisher’s test for analysis of variance (ANOVA) was performed. Further optimized terms of the independent variables applied to approve the model and to compare predicted results to the experimental data.

### 2.4. Determination of Total Phenolic Content

TPC assessed spectrophotometrically using Folin–Ciocalteu reagent [32]. The total phenol content is determined by the equation (y = 10.56X + 0.0189, r^2^ = 0.997) of the calibration curve of gallic acid and expressed in mg/100 g, the equivalent of gallic acid for the dry raw material. The absorbance was measured using a Genesys-10 UV/Vis spectrophotometer (Thermo Spectronic, Rochester, NY, USA), at 765 nm wavelength.

### 2.5. Determination of Total Proanthocyanidins Content

Spectrophotometric measurements were scored using a Genesys-10 UV/Vis spectrophotometer (Thermo Spectronic, Rochester, NY, USA). Total proanthocyanidins were determined by applying the technique described by [33]. Three mL DMCA solution (0.1% 4-dimethylamino cinnamaldehyde in methanol—HCl 9:1 *v*/*v*) was mixed with 20 μL of the extract. A decrease in absorbance was determined at a wavelength of 640 nm after 5 min. The concentration of condensed tannins in the extract was calculated based on a calibration curve established with catechin as a standard (calibration curve: catechin (mg/100 g) = (y − 0.0066)/3.1312), r^2^ = 0.995.

### 2.6. Antiradical Activity

The DPPH * free radical scavenging activity was determined using the slightly modified spectrophotometric method described by [34]. Two mL DPPH (2,2-diphenyl-1-picrylhydrazyl) solution in 99.0% *v*/*v* ethanol was mixed with 20 μL of the extract. A decrease in absorbance was determined at a wavelength of 515 nm after storing the samples in the dark for 30 min at a ambient temperature. An ABTS + radical cation decolorization assay was applied according to the methodology described by [35]. Then, 2 mL of ABTS (2,2′-azino-bis (3-ethylbenzthiazoline-6-sulphonic acid)) solution (absorbance 0.800 ± 0.02) was mixed with 20 μL of the extract. A decrease in absorbance was measured at a wavelength of 734 nm after storing the samples in the dark for 30 min. Results were expressed in μmol of Trolox equivalents in 100 g of dry extract and were calculated based on a calibration curve established using Trolox (6-hydroxy-2,5,7,8-tetramethylchromane-2carboxylic acid).

### 2.7. Determination of Ascorbic Acid (Vitamin C) Content

Ascorbic acid (vitamin C) was measured by AOAC’s (Association of Official Analytical Chemists) official titrimetric method (AOAC, 1990) [36].

### 2.8. Determination of Total Fibre Content

The total fiber content was determined using the enzymatic-gravimetric method, according to AOAC 985.29, 1997 [37].

### 2.9. High Performance Liquid Chromatography (HPLC) Method for the Determination of Phenolic Compounds

A Waters e2695 chromatograph equipped with a Waters 2998 photodiode array detector (Waters, Milford, MA, USA) was used for the HPLC analysis according to the methodology described by [38]. Chromatographic separations were carried out by using an YMC-Pack ODS-A (5 µm, C18, 250 × 4.6 mm i.d.) column equipped with a YMC-Triart (5 µm, C18, 10 × 3.0 mm i.d.) pre-column (YMC Europe GmbH, Dinslaken, Germany). The column operated at a constant temperature of 25 °C. The injection volume was 10 µL. The flow rate 1 mL/min, and gradient elution was used. The mobile phase consisted of solvent A-2% (*v*/*v*) acetic acid in water and solvent B-acetonitrile 100% (*v*/*v*). The following conditions of elution were applied: 0–30 min, 3–15% B; 30–45 min, 15–25% B; 45–50 min, 25–50% B; and 50–55 min, 50–95% B. The identification of the chromatographic peaks was achieved by the retention times and spectral characteristics (λ = 200–400 nm) of the eluting peaks with those of the reference compounds.

### 2.10. Preparation of Extracts for Antibacterial Testing

Twenty grams of freeze-dried quince fruit powder was mixed with 200 mL of 50% ethanol and extracted at the optimized condition. The extracts were filtered and dried in a rotary vacuum evaporator Büchi R-250, (Büchi Laboratortechnic, Flawil, Switzerland) to remove ethanol and later in a freeze-dryer ILShin FD 85125 (Ilshin Lab., Nam-myun, Yangju-si Gyeonggi-do, Korea) to remove the water. Dry extracts were kept in a freezer in hermetically sealed containers until used. Dry extracts were re-dissolved in 80% methanol to produce 0.5%, 1%, and 5% solutions, which were tested against microorganisms. The bacteria used in this study were stored at Micro-Bank (Pro-Lab Diagnostic, England) at −72 ± 3 °C before the start of the experiments. The bacteria were revitalized in the brain heart infusion broth (BHI, Oxoid, England) for 24 h, at the optimum temperature (30 ± 1 °C or 37 ± 1 °C). *B. subtilis* ATCC 6633 were grown on TSA (Liofilchem, Italy) agar slants for 24 h, at 30 °C. *Enterococcus faecalis* (ATCC 29212), *Staphylococcus aureus* (ATCC 25923), *Escherichia coli* (25922 ATCC), *Pseudomonas aeruginosa* (27853 ATCC), *Salmonella enterica serovar Typhimurium* (ATCC 14028) were grown on TSA agar slants for 24 h at 37 °C. *C. albicans* were grown on Sabouraud dextrose Liofilchem, (LD 610103) agar slants for 24–48 h at 25 °C.

### 2.11. Antimicrobial Activity Assay

The antimicrobial properties were evaluated by the agar well diffusion method according to the method described by [39]. The bacteria were grown in peptone-soy bouillon (LAB 04, LAB M) for 24 h at 37 °C. After cultivation, culture cells were mixed using a mini shaker MS 1 (Wilmington, NC, USA.) and the cell suspensions were adjusted according to McFarland nr 0.5 standard [40]. The cell suspensions of *C. albicans* were adjusted according to McFarland nr 1.0 standard. Then, 1 mL of the suspension of bacteria cells was introduced into 100 mL dissolved plate count agar *Liofilchem* (LD 610040), medium cooled to 47 °C. Then, 10 mL of the suspension was added into a 90-mm diameter Petri plate, the final concentration of cells in 1 mL was 1.5 × 10^6^. Eight mm diameter wells in agar were filled with 50 µL of extracts. The plates were incubated overnight at 37 °C. *B. subtilis* 30 °C, *C. albicans* 25 °C, in Sabouraud dextrose agar, Liofilchem, (LD 610103). Then, the inhibition zones were measured with calipers to an accuracy of 0.5 mm. As a control in the blank sample, aqueous methanol (80%) was used.

### 2.12. The Statistical Methods

All the experiments repeated three times and the results were expressed as means ± SD. Data were submitted to the analysis of variance (ANOVA). Tukey’s HSD (honest significant difference test) was used to evaluate the significant differences (*p* ≤ 0.05) between means (multiple comparison test). The statistical analysis was performed using Statistica 10 software (StatSoft, Inc., Tulsa, OK, USA).

## 3. Results and Discussion

### 3.1. Selection of Extraction Parameters

Maceration extraction (ME) with pure acetone provided the lowest content of phenolic compounds (Table 1). The acetone has the lowest dielectric constant from the tested organic solvents, which proves that the lower the relative static permittivity, the extraction efficiency of TPC is weaker [22,23]. The extraction of TPC efficiency significantly improved, when the water concentration in acetone and ethanol increased. It has been reported in previous studies, that dual solvent systems are more efficient for TPC extraction [22,23]. In contrast, the higher water concentration with methanol had a negative impact on extraction, and the highest TPC obtained with pure methanol.

There was no significant difference between the highest TPC values, which was obtained with acetone 70% and ethanol 50%, so for all further extractions, the latter was chosen. The central composite design prepared using response surface methodology (RSM) to optimize the extraction condition of TPC (Table 2). Different combinations of parameters had a significant effect on TPC in JQ fruit extracts, ranging from 4522.6 to 6784.9 mg/100 gDW (Table 2).

The suitability and significance of design was evaluated using the analysis of the variance (ANOVA), shown in (Table 3). A linear relationship was considered in our analysis, with the selected model (*p*-value = 0.0022). The results of the analysis also showed that only temperature (X_3_) had a significant effect on phenols extraction (*p*-value = 0.0003).

**Figure 1:**
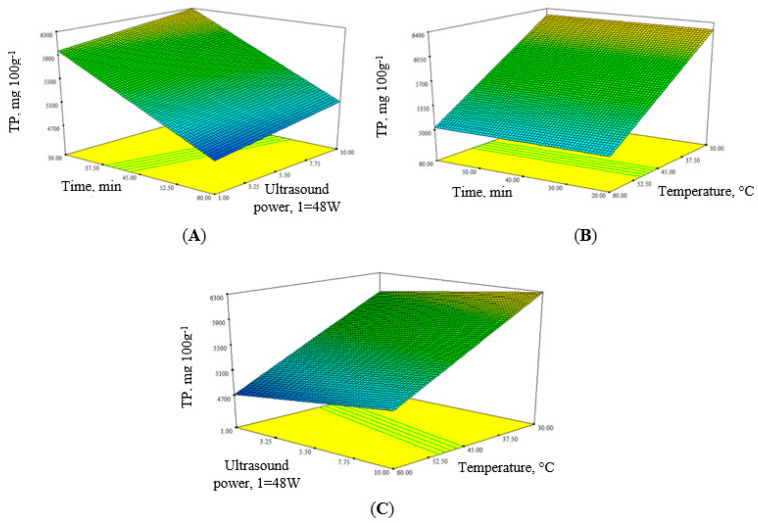
Response surfaces plots for total phenols (TP) in Japanese quince fruit, in the function of ultrasound power, extraction time, and temperature. (**A**)—Extraction time and ultrasound power; (**B**)—extraction time and temperature; (**C**)—extraction temperature, and ultrasound power.

To determine the most effective values of the variables, the three-dimensional surface plots (Figure 1), were designed according to the final predictive Equation (2), given below:

Response (TPC) = 5510.47 + 155.6*X*_1_ + 2.81*X*_2_ − 633.01*X*_3_. (2)

Figure 1A shows the overall response of extraction time, and ultrasound power to the TPC. The total phenols decreased when the extraction time extended and slightly increased when the ultrasonic power got stronger. This response shows that the long use of strong ultrasound has a negative effect on phenolic compounds.

Figure 1B,C confirms the analysis results that the temperature had a significant effect on the extraction efficiency, concerning both extraction time and ultrasonic strength. As the temperature raised, the phenols content decreased, as high temperatures caused degradation of compounds. To verify the model and the predicted amount of phenolic compounds (6296.3 mg/100 g), optimum extraction conditions were as follows: 20 min, at 30 °C with 480 W ultrasound power. The observed value obtained under these conditions was 6147 mg/100 g and the absolute error (AE) value 2.4%. Compared to simple maceration, ultrasonic extraction increased the phenol extraction from quince fruit by 14.5%, and the process time reduced from 24 h to 20 min.

### 3.2. Biochemical Composition and Antiradical Activity of Japanese Quince Fruit Extracts

The biochemical composition and antiradical activity of JQ fruits are presented in (Table 4). No significant difference was detected between cultivars using Folin–Ciocalteu assay for TPC, ranging from 3906 to 4550 mgGAE/100 gDW. Previous studies found a twice-lower TPC in JQ fruits; these differences may have been due to another extraction method, and that result was expressed in fresh weight [8].

However, the total content of proanthocyanidins (condensed tannins) differed significantly between cultivars, was lowest in ‘Rondo’ and highest in ‘Darius’ (Table 4). The total proanthocyanidins content accounts for 22–34% of the total polyphenol content. Proanthocyanidins have several types of bioactivities, e.g., antioxidant, cardio protective, neuroprotective, including antimicrobial activity [41]. The antiradical activity of the extracts with DPPH assay have shown a slight difference between cultivars and had a strong correlation with their TPC and proanthocyanidins content, r = 0.95, and 0.99, respectively. This is in agreement with previously provided studies, which demonstrated a strong correlation between the antiradical activity of berry fruits and their TPC [39,42]. A weaker correlation was found between antiradical activity by ABTS assay with TPC and proanthocyanidins content, r = 0.78, and 0.63, respectively. JQ fruits had a considerable amount of ascorbic acid (vitamin C), and appreciably similar to the previous study of nine JQ genotypes [1]. The total fiber content was not different between cultivars, averaged 30 g/100 g, and it is in agreement with the previously performed study of 12 JQ genotypes [12].

Using the HPLC method, five phenolic compounds identified: (–)-epicatechin, (+)-catechin, chlorogenic acid, rutin, and isoquercitrin (Table 5). The total flavan-3-ol (catechin and epicatechin) content accounts for around 94% of the total polyphenol content, indicating that they are the main polyphenol compounds in JQ fruits. Our results, that JQ fruits had more epicatechin, and less catechin and chlorogenic acid, coincided with previous studies [3,8].

No significant differences in TPC obtained by the HPLC method between cultivars. However, some differences were found between individual phenols, ‘Rasa’ had the highest amount of rutin, ‘Rondo’ and ‘Darius’ had more catechin and chlorogenic acid, respectively. There was a strong correlation between TPC using both Folin–Ciocalteu assay and HPLC method (r = 0.87), suggesting that TPC in JQ fruit was stable.

### 3.3. Antibacterial Activity

Our results show that all JQ cultivar possessed antimicrobial activity against three Gram-positive and three Gram-negative bacteria, and the most sensitive was *Enterococcus faecalis* (ATCC 29212) (Table 6). However, none of the extracts showed antifungal activity against *Candida albicans* yeast. Antibacterial activity of JQ extracts had a concentration-dependent manner, and the strongest inhibition effect was found using a 5% concentration (Table 6). As the concentration of the JQ extracts increased 10-fold, the inhibitory effect doubled. Extracts of ‘Rondo’ with 0.5% concentration has not inhibited the growth of *Staphylococcus aureus* (ATCC 25923), but the effect was due to the increased concentration. The lowest concentration extracts of all cultivars did not show any antibacterial effect on *Salmonella enterica serovar Typhimurium* (ATCC 14028), but 1% and 5% extracts inhibited this strain quite well. These results confirm previous studies, that extracts with higher phenols concentrations have stronger antibacterial activity [43,44].

The extracts of JQ cultivars more inhibited Gram-positive bacteria than Gram-negative, with very few exceptions. These differences probably depend on Gram-negative bacteria cells properties, which have an additional outer membrane with lipopolysaccharide molecules [45]. Besides, there are a number of reports that plant phenolic extracts have a stronger effect against Gram-positive bacteria [46,47,48]. Cultivar ‘Rasa’ had the strongest inhibitory activity against both Gram-positive and Gram-negative bacteria (Table 6). Interestingly, the fruit of the ‘Rasa’ had a significantly higher amount of rutin (Table 5). Other studies concluded that plant extracts, with higher levels of rutin, were more effective against bacteria [49,50]. In addition, rutin showed the ability to enhance the antibacterial activity of other phenols and antibiotics [51,52]. JQ fruit extracts had a stronger inhibitory effect against *E. coli* ATCC 25922 and *P. aeruginosa* ATCC 27853 than *S. aureus* ATCC 25923 [20]. The results of our study showed that only ‘Rasa’ extracts have similar activity, but ‘Darius’ and ‘Rondo’ extracts had the opposite effect, stronger inhibits *S. aureus* (ATCC 25923).

Our results also showed that antimicrobial activity of the extracts against bacteria was in good correlation with their rutin content (r = 0.98, 0.94, 0.92, 0.69, and 0.69 for *S. aureus*, *E. coli*, *E. faecalis*, *B. subtilis*, and *P. aeruginosa*, respectively), and epicatechin content (r = 0.94 for *Salmonella* ssp.). A previous study had reported similar results, that *E. coli* is more sensitive to rutin, and *Salmonella* to epicatechin [53]. It was noticed that the antibacterial potent of each cultivar was significantly different on all tested bacteria, with a few exceptions. The cultivars ‘Rasa’ and ‘Darius’ extracts had similar activity on *Staphylococcus aureus* and *Salmonella* ssp., and ‘Rasa’ and ‘Darius’ only on the *Salmonella* ssp.

There is no doubt that TPC determines the antibacterial activity, however, no significant differences were detected between cultivars. Differences in efficiency were probably due to the distribution of individual phenols between cultivars. The mechanisms of action of antibacterial activity are unequal for individual phenolic compounds, and their combinations [54,55,56,57]. Besides, individual bacteria have different resistance mechanisms, which are based on their biological properties [16]. Nevertheless, all of the bacteria cultures tested were sensitive to JQ fruit extracts.

## 4. Conclusions

All three JQ cultivars (‘Rasa’, ‘Darius’, and ‘Rondo’) are rich in bio-compounds and showed an important antibacterial activity against all tested bacteria. A considerable amount of phenols, vitamin C, and fiber were determined. The chemical analysis of these cultivars showed the presence of five phenolic compounds, whose main major compound detected was epicatechin. Our study results showed that JQ extracts not only have strong antiradical activity but also could effectively fight against three Gram-positive and three Gram-negative bacteria. The inhibition zone varied between different concentrations of the extracts and between bacterial strains. The food complemented with freeze-dried powder of JQ fruit can enrich the diet with strong antioxidants, which are important in maintaining human health and help to prevent various diseases. In addition, due to their antibacterial activity, they are very promising as a substitute for chemical preservatives in the food and cosmetic industry. These results are significant and quite important, especially due to growing consumer interest for natural products free of chemical additives.

## Figures and Tables

**Table 1:** The total phenolic compounds in Japanese quince fruit, mg/100 gDW.

Solvent	Solvents Concentration [%]		
	100	70	50
Ethanol	4409 ± 25 ^c^	5104 ± 32 ^b^	5256 ± 19 ^a^
Methanol	5195 ± 34 ^a^	4984 ± 22 ^b^	4796 ± 27 ^c^
Acetone	3228 ± 61 ^b^	5426 ± 83 ^a^	5274 ± 52 ^a^

The different letters in the same line indicate statistically significant differences between the samples.

**Table 2:** Experimental design of three-level, three-variable central composite design for ultrasound extraction phenols from quince fruit extracts.

Test Set	X_1_, Ultrasonic Power (W)	X_2,_ Extraction Time (min)	X_3_, Temperature (°C)	Total Phenols mg/100 g
1	240 (0)	40 (0)	45 (0)	5365.5
2	240 (0)	40 (0)	45 (0)	5219.2
3	480 (+1)	20 (−1)	60 (+1)	4522.6
4	240 (0)	40 (0)	60 (+1)	5047.3
5	48 (−1)	60 (+1)	60 (+1)	4851.1
6	240 (0)	20 (−1)	45 (0)	5579.9
7	48 (−1)	40 (0)	45 (0)	5830.9
8	48 (−1)	20 (−1)	60 (+1)	4729.5
9	240 (0)	40 (0)	45 (0)	5417.3
10	240 (0)	40 (0)	45 (0)	5866.3
11	240 (0)	40 (0)	45 (0)	5840.6
12	240 (0)	60 (+1)	45 (0)	5095.9
13	48 (−1)	60 (+1)	30 (−1)	6061.7
14	240 (0)	40 (0)	30 (−1)	6236.9
15	240 (0)	40 (0)	45 (0)	5007.3
16	480 (+1)	60 (+1)	60 (+1)	4785.2
17	480 (+1)	20 (−1)	30 (−1)	6435.2
18	48 (−1)	20 (−1)	30 (−1)	5515.3
19	480 (+1)	60 (+1)	30 (−1)	6016.7
20	480 (+1)	40 (0)	45 (0)	6784.9

**Table 3:** Analysis of variance (ANOVA for response surface linear model) showing the effect of the three independent variables on the extraction efficiency of phenolic compounds from quince fruit.

Source	Sum of Squares	df	Mean Square	F Value	*p*-Value
Model	4.25 × 10^6^	3	1.42 × 10^6^	7.6519	0.0022
Ultrasonic power	2.42 × 10^5^	1	2.42 × 10^5^	1.30814	0.2696
Extraction time	78.961	1	78.961	4.27 × 10^−4^	0.9838
Temperature	4.01 × 10^6^	1	4.01 × 10^6^	21.64713	0.0003
Lack of Fit	2.38 × 10^6^	11	2.16 × 10^5^	1.853435	0.2570
Pure Error	5.83 × 10^5^	5	1.17 × 10^5^		

**Table 4:** The biochemical composition and antiradical activity of Japanese quince fruits.

Properties	Japanese Quince Cultivars		
	‘Darius’	‘Rondo’	‘Rasa’
TPC, mgGAE/100 g	4550 ± 394 ^a^	3906 ± 77 ^a^	4366 ± 385 ^a^
Content of proanthocyanidins, mg/100 g	1550.1 ± 31.4 ^a^	879.7 ± 20.1 ^c^	1233.4 ± 15.6 ^b^
RSA (DPPH), µmol TE/100 gDW	115.9 ± 5.9 ^a^	99.1 ± 2.2 ^b^	106.5 ± 2.3 ^a^
RSA (ABTS), µmol TE/100 gDW	559.7 ± 34.2 ^b^	372.0 ± 5.0 ^c^	681.6 ± 11.7 ^a^
Ascorbic acid (vitamin C), mg/100 g	168 ± 2.1 ^a^	169 ± 1.1 ^a^	114 ± 2.8 ^b^
Total fiber content, g/100 g	28.5 ± 2.5 ^a^	28.5 ± 3.0 ^a^	31.2 ± 3.2 ^a^

The different letters in the same line indicate statistically significant differences between cultivars (*p* < 0.05). Results represent means ± SD (*n* = 3).

**Table 5:** The quantitative composition of phenolic compounds in quince fruit, µg/g DW.

Compound, µg g^−1^ DW	Japanese Quince Cultivars		
	‘Darius’	‘Rondo’	‘Rasa’
Isoquercitrin	38.8 ± 2.5 ^a^	33.3 ± 3.2 ^b^	42.4 ± 1.9 ^a^
Rutin	37.8 ± 3.4 ^c^	57.4 ± 2.2 ^b^	66.7 ± 2.2 ^a^
(+)-Catechin	131.8 ± 6.1 ^c^	182.9 ± 8.3 ^a^	157.9 ± 10.2 ^b^
(–)-Epicatechin	3535.1 ± 60.2 ^a^	3343.1 ± 55.1 ^b^	3575.9 ± 50.5 ^a^
Chlorogenic acid	152.9 ± 7.1 ^a^	98.6 ± 6.3 ^c^	113.7 ± 4.4 ^b^
Total	3896.3 ± 65.2 ^a^	3715.2 ± 58.1 ^a^	3956.7 ± 53.7 ^a^

The different letters in the same line indicate statistically significant differences between the individual compounds in the samples (*p* < 0.05).

**Table 6:** Antibacterial activity of Japanese quince fruit extracts.

	Microorganism	Extract Concentration, %	Japanese Quince Cultivars		
			‘Rasa’	‘Darius’	‘Rondo’
			Inhibition Zone Size, mm		
**Gram-positive**	*Bacillus subtilis* (ATCC 6633)	0.5	11.0 ± 0.1	11.0 ± 0.1	11.7 ± 0.5
		1	14.0 ± 0.1	12.0 ± 0.1	12.7 ± 0.4
		5	21.7 ± 0.5	18.3 ± 1.2	18.0 ± 0.1
	*Enterococcus faecalis* (ATCC 29212)	0.5	13.0 ± 0.1	12.0 ± 0.1	17.7 ± 0.4
		1	17.0 ± 0.1	15.0 ± 0.1	20.6 ± 0.5
		5	30.7 ± 0.5	25.3 ± 1.3	27.0 ± 0.2
	*Staphylococcus aureus* (ATCC 25923)	0.5	9.0 ± 0.1	9.0 ± 0.1	0
		1	12.0 ± 0.1	10.0 ± 0.1	13.7 ± 0.5
		5	18.7 ± 0.4	17.3 ± 1.1	18.0 ± 0.1
**Gram-negative**	*Escherichia coli* (25922 ATCC)	0.5	9.0 ± 0.1	10.0 ± 0.1	9.7 ± 0.5
		1	13.0 ± 0.1	12.0 ± 0.1	12.6 ± 0.5
		5	19.6 ± 0.5	15.3 ± 1.1	17.0 ± 0.1
	*Pseudomonas aeruginosa* (27853 ATCC)	0.5	9.0 ± 0.1	9.0 ± 0.1	9.6 ± 0.4
		1	13.0 ± 0.1	12.0 ± 0.1	11.7 ± 0.5
		5	19.7 ± 0.5	16.3 ± 1.1	16.0 ± 0.1
	*Salmonella enterica serovar Typhimurium* (ATCC 14028)	0.5	0	0	0
		1	12.0 ± 0.1	12.0 ± 0.1	11.7 ± 0.5
		5	19.7 ± 0.4	17.3 ± 1.2	15.0 ± 0.1
	*Candida albicans* (ATCC 10231)	0.5	0	0	0
		1	0	0	0
		5	0	0	0

Results represent means ± SD (*n* = 3).
